# Causal Inference for Spatial Constancy across Saccades

**DOI:** 10.1371/journal.pcbi.1004766

**Published:** 2016-03-11

**Authors:** Jeroen Atsma, Femke Maij, Mathieu Koppen, David E. Irwin, W. Pieter Medendorp

**Affiliations:** 1 Radboud University, Donders Institute for Brain, Cognition and Behaviour, Nijmegen, The Netherlands; 2 University of Illinois at Urbana-Champaign, Department of Psychology, Champaign, Illinois, United States of America; University of Birmingham, UNITED KINGDOM

## Abstract

Our ability to interact with the environment hinges on creating a stable visual world despite the continuous changes in retinal input. To achieve visual stability, the brain must distinguish the retinal image shifts caused by eye movements and shifts due to movements of the visual scene. This process appears not to be flawless: during saccades, we often fail to detect whether visual objects remain stable or move, which is called saccadic suppression of displacement (SSD). How does the brain evaluate the memorized information of the presaccadic scene and the actual visual feedback of the postsaccadic visual scene in the computations for visual stability? Using a SSD task, we test how participants localize the presaccadic position of the fixation target, the saccade target or a peripheral non-foveated target that was displaced parallel or orthogonal during a horizontal saccade, and subsequently viewed for three different durations. Results showed different localization errors of the three targets, depending on the viewing time of the postsaccadic stimulus and its spatial separation from the presaccadic location. We modeled the data through a Bayesian causal inference mechanism, in which at the trial level an optimal mixing of two possible strategies, integration vs. separation of the presaccadic memory and the postsaccadic sensory signals, is applied. Fits of this model generally outperformed other plausible decision strategies for producing SSD. Our findings suggest that humans exploit a Bayesian inference process with two causal structures to mediate visual stability.

## Author Summary

During saccadic eye movements, the image on our retinas is, contrary to subjective experience, highly unstable. This study examines how the brain distinguishes the image perturbations caused by saccades and those due to changes in the visual scene. We first show that participants made severe errors in judging the presaccadic location of an object that shifts during a saccade. We then show that these observations can be modeled based on causal inference principles, evaluating whether presaccadic and postsaccadic object percepts derive from a single stable object or not. On a single trial level, this evaluation is not “either/or” but a probability that also determines the weight by which pre- and postsaccadic signals are separated and integrated in judging object locations across saccades.

## Introduction

During saccadic eye movements, the image of the world shifts across our retina. Despite these shifts, we perceive targets as having world-stable positions, and have no problem to act upon them whenever necessary. It has been suggested that a combination of predictive and feedback mechanisms subserve this faculty, referred to as spatial constancy [1].

In the literature, spatial constancy has been studied by using motor and perceptual tasks. Using motor tasks, it has been shown that we can look or reach accurately to the remembered position of a target after an intervening saccade (see [1] for review). Using arm movements, Vaziri et al. [2] recently tested the hypothesis that the brain computes the position of a reach target after a saccade based on the optimal integration of predicted and actual sensory feedback. In their paradigm, participants first made a saccade after they briefly foveated a visual target in complete darkness. The brain is known to predict the new retinal position of this target after the saccade by internally remapping its representation relative to gaze [1, 3, 4]. Next, the target was postsaccadically viewed for a variable duration, slightly displaced relative to its initial position, before the participant reached at it. Results show that reach endpoints had smaller variance than was possible based on the predicted (i.e. remapped) estimate or the actual postsaccadic estimate alone, consistent with integration. The authors further demonstrated that the uncertainty of the postsaccadic target position, which was modulated by varying its viewing time, affected its weight in the integration process.

From a perceptual perspective, it has been shown that the sensitivity to perceive the displacement of a visual target severely drops during a saccade. In fact, target displacements up to one third of the saccade amplitude typically go unnoticed, which is known under the term saccadic suppression of displacement (SSD; e.g. [5]). Remarkably, blanking the target briefly after the saccade, before it reappears at a displaced position, significantly improves the sensitivity to the displacement [6], as does merely changing some characteristic of the saccade target, such as its form or polarity [7, 8]. This has led to the notion that the visual system a priori assumes that a target will not move or change during the saccade. If this assumption is broken, as with the blank, form change, or with large displacements, it causally regards the postsaccadic target as a new object, and computes the old position using retinal and extraretinal signals.

Niemeier et al. [9] formulated the SSD findings from an optimal integration perspective by combining visuomotor signals with a prior that reflects the assumption that targets are not displaced during the saccade. As predicted by their model, behavioral reports show that SSD has a nonlinear relationship with the size of the target displacement. While with small displacements the localization of the initial presaccadic target was strongly contracted to the postsaccadic target, this integration effect was reduced with larger displacements, making localization more veridical.

But how does the brain know when to integrate signals and when to process them independently in the computations to obtain spatial constancy? From a perceptual viewpoint, Vaziri et al. [2] essentially used a blanking paradigm, thereby ignoring the possible assumption that visual targets typically do not move during saccades. Despite the blank, which is assumed to indicate that sources are unrelated, their results show optimal integration of the presaccadic target information and actual postsaccadic target position. Also in the model of Niemeier et al. [9], the spatial constancy computations are unconditioned to causality: integration always occurs even with large target displacements.

In the present study, we test the role of causal inference in the computations to obtain spatial constancy. According to this framework, the brain has to estimate the causal relationship between the presaccadic and postsaccadic signals to establish to what degree they can be integrated or when they should be kept apart, which not only depends on the precision of these signals but also on their spatiotemporal difference [10, 11]. Based on the presaccadic input, it could be hypothesized that initially foveated representations are less susceptible to SSD than non-foveal representations because their remapped representations are more precise, triggering a segregation strategy. Based on the postsaccadic input, it could be proposed that if the postsaccadic target is presented only briefly, its representation is too weak to infer a target displacement, making the brain rely most heavily on an integration strategy in the later localization of the target. But if the postsaccadic target is viewed longer, displacements may become better detectable, triggering a segregation strategy, especially with large displacements.

Here, we test these hypotheses by varying the duration of the postsaccadic display in an SSD task for displacements of the initial fixation target, the saccade target and a non-foveated peripheral target. Because previous studies reported direction-specific SSD, (e.g.[12, 13]) we test for both parallel and orthogonal displacements relative to the direction of the saccade.

We show that spatial constancy is not based on the exclusive integration of presaccadic target information and actual postsaccadic sensory feedback nor does it follow from an a-priori assumption that targets do not move during saccades. Our results suggest that spatial constancy naturally follows from the principles of causal inference involving two possible causal structures: one where the pre- and postsaccadic percepts represent the same stable object (i.e. have a common cause), and one where two distinct objects are perceived (i.e. no common cause).

## Results

**Fig 1:**
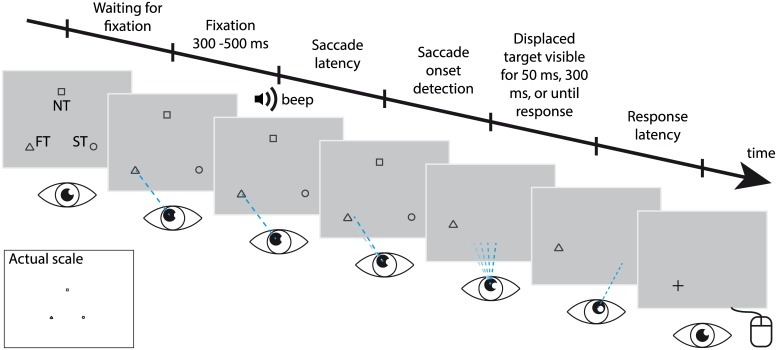
Graphic representation of a trial. Each trial started with the presentation of three objects, of which the FT (here: triangle) was foveated. After an auditory go cue, a horizontal saccade was initiated to the ST (here: circle). Upon detection of the saccade, two objects were removed from the screen while the other was displaced (here: orthogonal). The displaced object remained visible for 50 ms, 300 ms or until the response was given (~1000 ms). The remembered presaccadic location of this object was indicated using a computer mouse.

Participants were tested in a saccadic suppression of displacement task in which they had to indicate the presaccadic position of either the fixation target, the saccade target or a peripheral non-foveated target that was displaced parallel or orthogonal during a horizontal saccade (Fig 1). The displaced target was subsequently viewed for three different durations (50, 300 or ~1000 ms).

**Fig 2:**
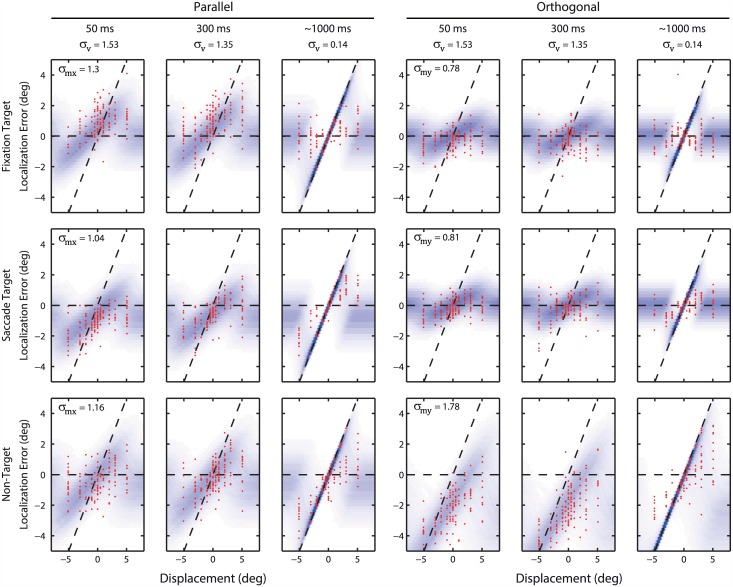
Performance of a single participant. Red dots represent localization responses and blue shaded areas represent the response probabilities, *p*(*s*|*mv*), according to the best-fit model predictions. Left three columns, localization errors for parallel target displacements; right three columns, errors for orthogonal displacements. Horizontal dashed line represents veridical localization, i.e. the segregation strategy. Dashed diagonal line represents the displacement of the postsaccadic target. With small displacements, errors deviate toward the diagonal line for the three targets; this pulling effect appears stronger with longer viewing durations.

Fig 2 shows the performance of a typical participant, plotting the localization errors (red dots) of the three target positions (rows: FT, ST, NT) as a function of parallel and orthogonal target displacement, respectively, separately for the three postsaccadic viewing times. Blue shaded areas represent best-fit model predictions, and will be discussed below. Data points should fall along the horizontal dashed line if the participant correctly remembered the presaccadic target location and ignored the target displacement after the saccade. In contrast, if the position of the postsaccadic target (dashed diagonal line) interacts with memory for the presaccadic position of the target, the data should diverge from the horizontal line and linearly relate to the size of the target displacement.

The localization responses of this participant indicate a mixture of these two patterns. While localization errors become larger with increasing target displacements, beyond a certain target displacement they transition back to smaller errors. Thus, with increasing target displacement, there appears to be a shift in the proportion of responses that are contracted to the postsaccadic target vs. the ones that are unaffected by it. This pattern can be seen in all panels.

**Fig 3:**
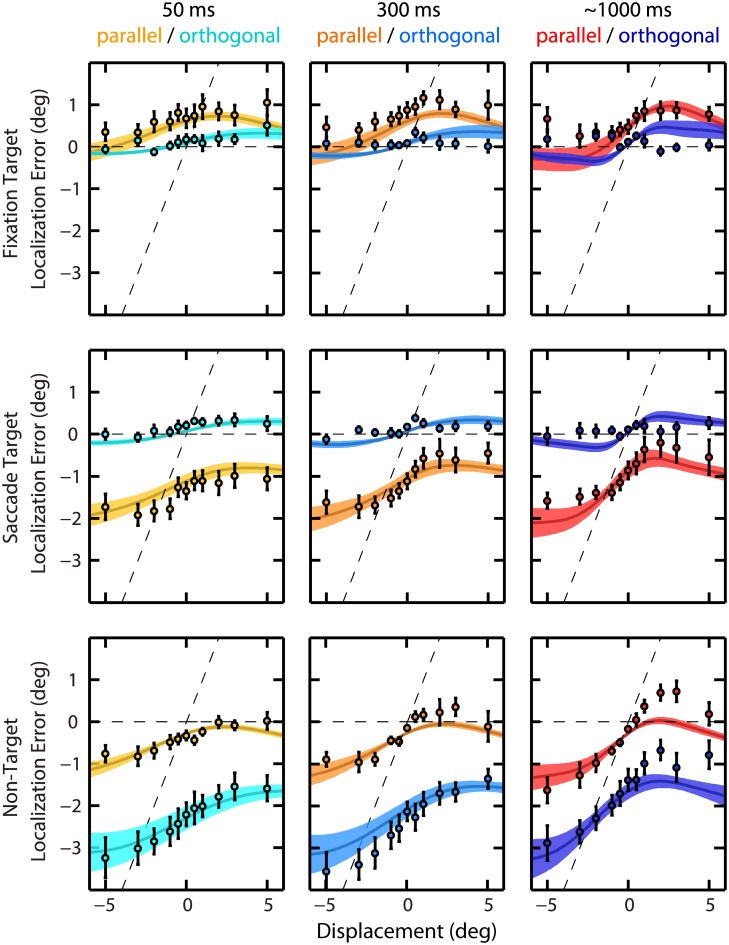
Mean localization errors across participants. Mean responses are shown as dots (error bars, SEM) and mean model fits as continuous lines (shaded areas, SEM). Format as in Fig 2.

Fig 3 depicts the localization errors, averaged across participants. The pattern of localization errors is similar to the results of the single participant shown in Fig 2, particularly the bias toward the postsaccadic target for small displacements and the loss of this contraction for large displacements. Below, this will be interpreted as the outcome of a mixture model balancing integration and segregation processes, but this qualitative structure can already be confirmed by standard statistical analysis. The distinction between small and large displacements is not a sharp one, of course, and could, in a functional sense, depend on target position, viewing time and direction of displacement. Therefore, we took for the following analyses the displacements with absolute value strictly smaller than 2° (0, ±0.5, ±1°) as “small” and the displacements with absolute values strictly greater than 2° (±3, ±5°) as “large”. (Replicating the analyses with the ±2° displacements added to either the “small” or “large” group turned out to yield very similar results.)

An analysis including the three targets (FT, ST, NT), the three viewing times (50, 300, ~1000 ms), the two directions (parallel, orthogonal) and the “small” displacements (0, ±0.5, ±1°), showed a significant positive linear effect of displacement on localization error (*F*(1,10) = 28.7, p < .001). This effect was present across targets, viewing times and directions, but it was moderated by these factors. For instance, post hoc comparisons revealed that the regression slope of localization error on displacement was less steep for FT trials than for ST and NT trials, the latter two not differing significantly. This is in line with the notion that because FT is initially foveated, it is represented more precisely than ST and NT, and therefore less influenced by its postsaccadic location. As for viewing time, the slope was generally less steep for 50 than for 300 ms, with no significant difference between 300 and ~1000 ms. Overall, parallel displacements produced a steeper slope than orthogonal ones. The moderating effects of viewing time and direction, however, were not present for all targets (a 2^nd^ order interaction). For the FT, slopes were not significantly different across viewing times and directions, although they tended to be steeper for parallel than orthogonal displacements (*p* = 0.06). For ST trials, there was no moderating effect of time, but a very clear effect of direction (*p* < .001), with a steeper slope for parallel displacements. In contrast, the NT trials showed no moderation of the slope by direction, but they did show a very clear effect of time: here the slope was significantly steeper for ~1000 ms than for 300 ms (*p* = .018), as well as for 300 ms compared to 50 ms (*p* = .025). All in all, this makes for a complicated collection of results, which have in common across all conditions, however, a positive linear effect of small displacements on localization error.

This linear relationship between displacement and localization error does not extend to the large displacements. Choosing either the positive large displacements 3° and 5°, or the negative displacements -3° and -5° revealed no effect of displacement on localization error (*p* = .87 and *p* = .28, respectively) in an analysis including the target, viewing time, and direction factors.

To explain these effects, we modeled the role of causal inference in the computations to obtain spatial constancy. Our principal model involves a statistically optimal mixture at the trial level of two possible causal structures on the signals available (see Methods). For each participant, the model was fit to all localization errors simultaneously. For the participant in Fig 2, the best-fit model is shown by the blue shaded curves. The shade intensity represents the model’s likelihood of localization errors (*p*(*s*|*mv*)). The model adequately predicts the positive slope in the errors as observed with small but increasing target displacements. This positive slope reflects the model’s weight on the assumption that the pre- and postsaccadic percepts originate from the same stable target (i.e. have a common cause), so they can be integrated to estimate a more precise but biased response (in the direction of the postsaccadic target). Along the same lines, the model also accounts for the effects of postsaccadic viewing time, the increase of which causes a more precise postsaccadic representation resulting here in a steeper slope in the localization error (i.e. a stronger contraction or pull to the postsaccadic target). Finally, the model infers that for large target displacements, the pre- and postsaccadic percepts likely stem from different causes, for which it is optimal to not integrate but rather disregard the postsaccadic percept. As a result, the probability of a localization response toward the displaced target decreases, which matches with the transition to smaller errors as observed in the data.

The continuous lines in Fig 3 depict the best-fit predictions from the model, averaged across participants. As shown, these curves display a good correlation with the localization errors (*R*^2^ = .65 ± .06 and *R*^2^ = .85 ± .03 for the parallel and orthogonal direction, respectively, across participants; see the section Mixture Model for details about the fitting procedure).

**Fig 4:**
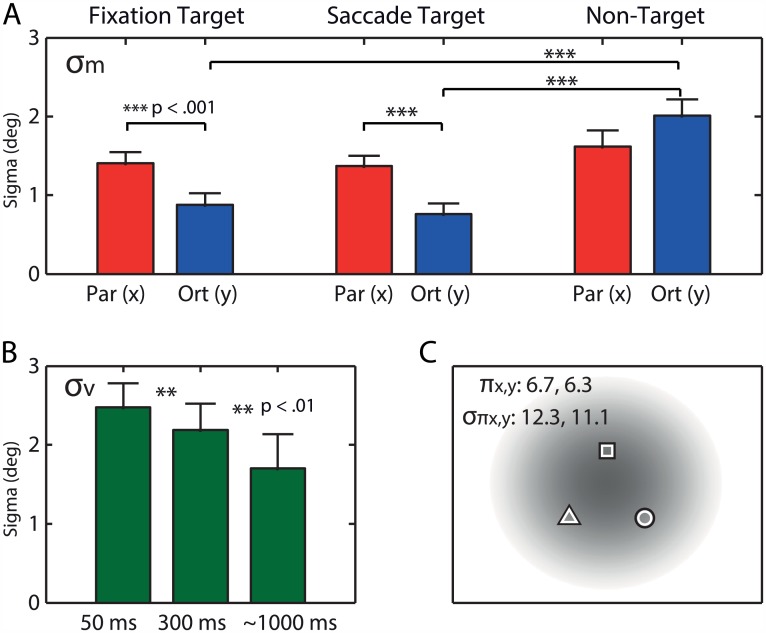
Mean parameters of best-fits. (A) Average *σ*_*m*_ across participants (error bars, SEM). The orthogonal component of the memorized positions appears to be more precise than the parallel component for FT and ST, but not for NT. (B) Average *σ*_*v*_across participants. Variability of the postsaccadic-target representation decreases as a function of viewing duration. (C) Prior π, positioned relative to FT, representing where objects are generally expected to appear. All values are in degrees.

The best-fit parameter values (see Table 1) give insight in the precision with which the target positions are recovered from memory when computing the localization responses (*σ*_*m*_; see Fig 4A). A two-way analysis on the *σ*_*m*_ values revealed significant effects of both target (*F*(2,9) = 31.9, *p* < .001) and displacement direction (*F*(1,10) = 5.2, *p* = .045), as well as a significant interaction effect (*F*(2,9) = 25.9, *p* < .001). The interaction is expressed by the finding that this effect is mostly driven by the orthogonal displacements (see Fig 4A). Post hoc comparisons revealed that NT is memorized with a lower precision than FT and ST. Thus, while both ST and NT are viewed in the periphery before the saccade, ST is memorized with higher precision than NT. No significant difference was found between the estimated parameters for FT and ST.

**Table 1:** Best-fit parameter values for all eleven participants. All values are in degrees except probability P_C_. Position of π is expressed relative to FT.

*σ*_*mx*_			*σ*_*my*_			*σ*_*v*_								
FT	ST	NT	FT	ST	NT	50	300	1000	*σ*_*f*_	*σ*_*πx*_	*σ*_*πy*_	*π*_*x*_	*π*_*y*_	*p*_*c*_
1.56	1.65	2.49	1.08	1.32	2.86	3.11	2.25	1.42	4.76	16.56	12.56	12.62	15.92	0.57
1.22	1.6	1.14	0.41	0.53	1.67	3.51	3.35	3.33	3.38	5.55	4.86	12.84	20.53	0.02
0.85	0.88	0.9	0.46	0.46	1.25	3.12	3.11	3.06	3.92	9.86	16.04	4.77	-0.53	0.78
0.9	1.05	1.24	0.88	0.69	1.45	1.39	1.17	0.07	4.29	19.36	11.12	9.22	0.5	0.09
1.3	1.04	1.16	0.78	0.81	1.78	1.53	1.35	0.14	3.66	17.82	11.28	1.11	12.23	0.21
0.87	0.98	1.16	0.49	0.38	1.27	3.39	3.23	3.1	4.57	3.61	20.3	7.65	2.43	0.62
1.68	0.83	1.02	0.62	0.48	2.27	0.9	0.47	0.26	6.65	23.93	14.29	6.1	-0.34	0.02
2.46	1.86	1.79	2.3	1.26	2.93	1.96	1.34	0.18	5.15	7.75	2.98	3.11	3.12	0.53
1.79	1.29	1.73	1.22	1.1	2.64	1.81	1.61	1.48	5.09	7.82	7.71	2.05	1.03	0.39
1.46	1.84	2.26	0.75	0.77	2.31	2.49	2.31	1.87	4.63	9.04	10.76	13.64	10.15	0.73
1.37	2.04	2.9	0.68	0.57	1.69	3.99	3.9	3.83	4.39	14.35	10.62	0.76	3.93	0.96

Fig 4B depicts the model’s prediction of the precision of the postsaccadic target (*σ*_*v*_) for the three viewing times. Here the effect of viewing time is significant (*F*(2,9) = 7.5, *p* = .012) and, as expected, post hoc comparisons reveal precision to improve (lower sigma values) both from 50 to 300 ms viewing (*p* = .004) and from 300 to ~1000 ms viewing (*p* = .008).

As the mean data show, there are also errors in the absence of any target displacement. The model explains this by the combined effect of the foveal prior (*σ*_*f*_ = 4.6° ± 0.27°, mean ± SEM) and the allocentric prior *π*. The location and precision of the allocentric prior are plotted in Fig 4C, showing that it is centered in between the three target locations, and has a substantial width (~12°) compared to the inferred precision vales of both the remapped, presaccadic target representations (Fig 4A) and postsaccadic information (Fig 4B).

Finally, in the model, the general degree by which participants’ localization responses were influenced by the displaced target is captured by parameter *p*_*c*_, which represents the prior probability that the target remains stable. Its value was on average 0.45 ± 0.1 (mean ± SEM), but Table 1 shows that this parameter varied substantially among the 11 participants. This prior in combination with the information of *m* and *v*, results in a posterior probability that the target has not moved, *p*(*C*|*mv*), as a function of target displacement.

**Fig 5:**
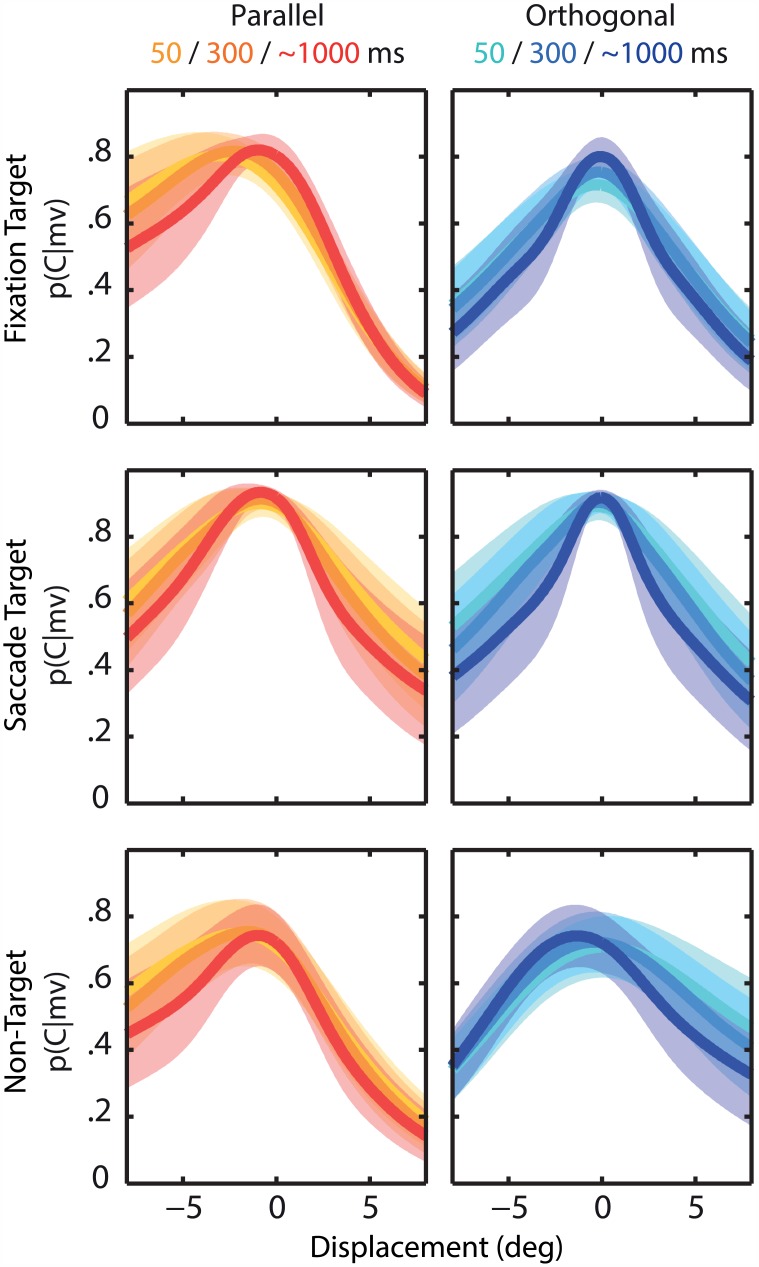
Inferred probability of a common cause *p(C|mv)* as a function of target displacement. Probabilities are based on the best-fit parameters, separated by target location (rows: FT, ST, NT), displacement directions (columns: parallel/orthogonal) and postsaccadic viewing duration(in color). Shown are the mean values across participants and standard error (shaded areas). This probability, which can be interpreted as the complementary probability of perceiving the displacement, optimally weights the integration and segregation strategy.

Fig 5 shows that the average *p*(*C*|*mv*) is close to one for small displacements, suggesting integration of pre- and postsaccadic targets. For larger target displacements, the curves fall off, suggesting more evidence that pre- and postsaccadic representations stem from different sources. The curves also illustrate the effect of viewing time: when the postsaccadic target is viewed only briefly, inferring causality becomes more difficult, resulting in a more gentle decline of *p*(*C*|*mv*) with increasing displacements.

The above results follow from fits of a mixture model that assumes a causal inference process that is fully statistically optimal. For comparison, we also fitted two variants of this model, model selection and probability matching (see Methods). The models differ by the response rule applied (see Methods). Across our participants, on average the log-likelihood differences of these models with the mixture model were 344 ± 124 and 125 ± 49, respectively, indicating that the mixture model (average log-likelihood -17262) outperforms its variants. Since the three models share the same parameters, using an AIC or BIC instead of the log-likelihood criterion in the model comparison would not change this conclusion. For one participant (number 6) no clear difference between the mixture model and model selection was found (log-likelihood difference < 3); for two other participants (number 9 and 11), a probability matching strategy was ranked before the mixture model.

## Discussion

In the current study we modeled and tested the role of causal inference in the computations for spatial constancy across saccades. According to our model, the brain has to estimate whether presaccadic and postsaccadic signals reflect a stable or an unstable visual world, which depends on the spatiotemporal difference between these signals and on their precision. We operationalized the problem experimentally by using the saccadic suppression of displacement paradigm. Participants viewed three targets, with one of them the fixation point, the other the saccade target and the third a peripheral target. After the saccade, one of these three remained for different viewing durations, but often at a slightly displaced position, and participants had to indicate which location it had prior to the saccade. Our results show that: 1. the integration of the pre- and postsaccadic target positions declines as a function of their spatial separation, 2. different targets show different strengths of SSD, and 3. viewing time of the postsaccadic target changes the strength of SSD. Our model could account for all these findings, which will now be discussed in more detail.

We replicated the non-linear localization response pattern previously reported by Niemeier and colleagues [9], but modeled it in a different way. Sensory signals are inherently noisy. This means that even in the case of a completely stable world the pre- and postsaccadic percepts may show some false discrepancy which should be ignored by the brain. In the model of Niemeier and colleagues a spatial window of stability is created by integrating a displacement vector (i.e. the visual discrepancy) with a prior centered at zero displacement. This predicts that localization is pulled to the postsaccadic target, irrespective of the size of the displacement. The present model goes a step further, and considers this pulling effect from a causal inference perspective, stating that presaccadic and postsaccadic percepts should be integrated when their discrepancy is relatively small but should be segregated when the displacement increases. More specifically, it infers the probability that a common cause underlies the pre- and postsaccadic percepts. The model dealt with these considerations in an optimal manner, i.e. on any trial it applied a mix of both integration and segregation, each weighted by its respective probability as based on the precision of both percepts, thereby minimizing quadratic error in the long run. Of course, there are alternative forms by which the brain could process the inference about the common cause (see [10]). For example, the brain could also select per trial which causal structure is most likely, and accordingly process the trial in a binary fashion either by integration or by segregation. In most participants, we found that our weighted averaging model better described the data than a model involving binary selection or a model based on the principle of probability matching.

In the comparison of the fits of the three models described, Wozny et al. [10] found the last and least optimal variant, probability matching, the clear winner in a multisensory perception experiment. It must be noted, however, that our experimental setting differs principally from that of Wozny et al. and of other applications of the mixture model known to us [11, 14]. They deal with multisensory perception, where bimodal cues (typically auditory and visual) are available to be combined if there is evidence they belong to the same object, even though each unimodal cue is in itself sufficient to solve the task (e.g., localize an object). Data for either unimodal condition (just the auditory cue or just the visual cue) can be obtained without changing the task. In our case, there are two complementary representations in one modality (vision) and a division in an experiment with “just the presaccadic remapped memory information” and one with “just the postsaccadic visual information” is not sensible. Consequently, the outcome of the model comparison might well be different for our case.

As predicted by our model, we found strong integration when the target displacements were small, characterized by low response variability but large biases toward the postsaccadic target. Increasing the size of the displacement lowers the probability of a common cause (Fig 5) which results in smaller localization errors (Fig 3). The inferred probability of a common cause can directly be interpreted as the strength of SSD. As shown previously (e.g. [15]), displacements up to one third of the saccade amplitude typically show strong SSD. However, we have found differences in the strength of SSD between targets and displacement directions.

We showed that the differences in strength of SSD between targets reflect differences in the precision of the presaccadic target representations upon recall. The regression analysis suggests that FT is represented more precisely than ST and NT, while the model fits showed that both FT and ST were represented more precisely than NT. We lack a clear explanation for this difference, but as shown in Fig 3, the model generally underestimates the pulling effect of ST and overestimates this for FT. For both FT and ST, localization is better with orthogonal than parallel target displacements, which can be explained by the anisotropy in the precision of their memories. This anisotropy may result from the noisy eye position signals that are used to remap the target representation across saccades [9]. Indeed, our participants showed about twice as much scatter in the saccade end points in the direction of the saccade than orthogonal to it (1.27 ± 0.05° and 0.73 ± 0.03°, respectively, mean standard deviation ± SEM). The estimated parameters of the mixture model indicate that memory precision of FT and ST is also about two times worse parallel than orthogonal (see Table 1), which suggests that noise sources related to eye position sense play a role in the coding of these representations [9]. The memory of NT, which we found to be less precise than ST and FT, appears to be more variable in the orthogonal than along the saccade direction. Although we cannot explain all the differences in the strength of SSD among the three targets, an important factor may relate to how the brain has coded the visual scene in memory, which we will discuss next.

It has been suggested that across saccades the brain stores a structural description of the target display in memory (e.g. [16]). For example, in a task where participants have to remember a pattern of dots, it was shown that the relative positions of the dots could be recalled independent of absolute spatial information [17]. After a saccade, the saccade target could serve as an anchor to which the structural description is related [15, 18, 19]. Connecting this finding to the present experiment suggests that participants encoded the equilateral triangle constituted by the three targets. In our experiment, however, the majority of trials had no ST present after the saccade. If the structural description of the target display would then be anchored to the eyes’ landing position instead, it would predict a positive relationship between the saccade landing error and localization error. Indeed, we found a small but significant correlation for ST in almost all participants (mean r = .18). In the same vein, this notion could also explain why the ST was recovered with higher precision from memory than NT although both were presaccadically presented at equal eccentricities. If participants indeed stored a structural description as an equilateral triangle, there may be some variability in the size of the triangle from trial to trial. This variability would bear out in more response variability in the orthogonal direction of NT, as we have found. Furthermore, previous work has shown that a group of random static dots are typically remembered closer to each other than they actually were [20, 21], like our participants did. Our model explains this observation using an allocentric prior, positioned at about the center of the target display, albeit with some variability among participants This is consistent with current models of efficient coding in visuospatial memory, which propose that people code a display in terms of summary characteristics, such as its center of mass (e.g. [22, 23]).

Despite relative coding accounts, as described above, there is also ample evidence that the brain keeps target representations in a dynamic register (for a review see [4]). These representations, coded in eye-centered coordinates, must be updated when the eyes move. In support, several brain regions have been identified that contain neurons with visual receptive fields (RFs) that are normally fixed to one position of the retina but briefly shift in anticipation of a planned saccade to the position the RF will occupy after the saccade (e.g. [3, 24, 25]). Although it is currently unknown how the brain transfers object information across shifts of the RFs, it could be an important mechanism in order to achieve space constancy (e.g. [26]). In our experiment, the three target representations would be shifted in the opposite direction of the upcoming saccade. After the saccade a one-to-one comparison can be made between the postsaccadic retinal input and the predicted input to assess visual stability. It could be hypothesized that in anticipation of a saccade a given receptive field shifts in the accurate direction but with a less accurate amplitude. This seems plausible given that saccades to a target typically show more variability in amplitude than direction. While this would be consistent with the observed SSD differences for FT and ST, this is not the case for the NT. VanRullen [27] has argued that while the visual world translates homogeneously during a saccade, its cortical representation does not because the amount of cortex dedicated to a certain sized patch of the retina varies, especially as a function of retinal eccentricity. One possibility is that these non-homogenous shift of RFs introduces noise orthogonal to the saccade in the periphery, which may explain our results for the NT. A precise mapping of shifting RFs would be needed to test this hypothesis.

Alternatively, one could speculate that the observed differences between target locations reflect distortions due to RFs that shift not in parallel but towards the ST in anticipation of a saccade [28, 29]. Although it has been suggested that this anticipatory transient increase in density of receptive fields around the saccade target underlies the boost in attention around the ST area, and thus is beneficial for space constancy for that target, it may be that the encoding of peripheral targets becomes distorted because of these RF shifts. The representation of a target like NT may become stretched or displaced towards the ST, resulting in a compressed memory. Future research should investigate whether these RFs do indeed distort perception.

In our experiment, we not only displaced the target but also manipulated the postsaccadic viewing time. In general, longer viewing increases its pulling effect on the localization response. Recently, Zimmerman et al. [30] performed a SSD task in which the viewing time of the presaccadic target was varied. They showed that when the presaccadic target is briefly viewed, i.e. < 0.5 s, displacement detection performance is low. Here, we modeled viewing time as a factor that changes the precision of the target representation. Indeed, the longer the target was visible, the higher its precision. In terms of our model, the viewing time manipulation by Zimmerman and colleagues would affect *σ*_*m*_ which in turn affects the probability of perceiving a common cause *p*(*C*|*mv*). In other words, the system is generally more likely to integrate when the representation of the presaccadic target is noisy, hence displacement detection performance is low. In our experiment, decreasing the viewing time of the postsaccadic target did generally lower the detection performance as well (i.e., increase *p*(*C*|*mv*)). The latter may not be directly obvious from the localization responses which show the strongest pulling effect with the longest viewing duration. The explanation is as follows. Although the integration strategy receives less weight with long viewing, the postsaccadic target representation is more precise, which has an opposite effect and ultimately pulls localization towards it.

A final point of discussion relates to model parameter *p*(*C*), which represents the a priori probability that the world remains stable. We found a considerable variability among participants for this parameter. In most participants, the *p*(*C*) estimates can be regarded low, given that in daily life objects rarely jump while we scan the world. We consider it plausible that the experimental context and task instruction, which explicitly mentions the possibility of displacements, alters *p*(*C*). For example, if you know beforehand that a certain scene will contain a lot of instability, it seems logical to lower *p*(*C*) and thus become more skeptical regarding the feasibility to integrate percepts.

Taken together, we showed that integration of the pre- and postsaccadic target representations can be modeled using principles of causal inference. When representations follow from spatially close target locations, integration is strong. In contrast, when targets are further apart, integration weakens, depending on precision of involved representations.

## Materials and Methods

### Ethics statement

The study was part of a research program approved by the local ethics committee of the Social Sciences Faculty of Radboud University (ECG2012-1304-030).

### Participants

Twelve naïve participants (eight females, average age 25.7 ± 0.6 years, mean ± SEM) participated in the experiment, all with normal or corrected-to-normal vision. Each participant participated in four experimental sessions of approximately 1 h each and informed consent was given beforehand. One participant did not complete all sessions because the eye-tracker helmet felt uncomfortable. We discarded her data.

### Experimental setup

Participants sat in a dimly lit room with their head supported by a chin rest. They operated a two-button computer mouse. Stimuli were controlled using a custom-written program in Delphi (Embarcadero) software. Visual stimuli were displayed on a 19 inch CRT monitor (Philips 109B) using a vertical refresh rate of 100 Hz and a resolution of 1024 x 768 pixels. The monitor was positioned about 30 cm in front of the participant’s eyes, encompassing 61° x 46° (HxV) of the visual field. A photodiode was placed over the bottom-left corner to determine the precise onset and displacement of the visual stimuli with respect to eye movements. Binocular eye position was recorded at 500 Hz using a head-mounted eye tracker (EyeLink II; SR Research). The eye tracker was calibrated using a 9-point grid. A saccade was detected online using a position threshold of 1.5°. Participants were allowed to take breaks every 400 trials. After each break the eye tracker was recalibrated and as needed during testing, for example when the program failed to detect a fixation at the start of a trial.

### Experimental protocol

We tested participants in an SSD task with three target positions, each of which contained a gray shape (circle, square, or triangle, all 1° size). Fig 1 presents a graphical depiction of a trial. At the start of the trial, the three target shapes appeared 15° apart at equilateral triangular positions against a light-grey background. The shapes designated the fixation target (FT), the saccade target (ST), and a peripheral non-target (NT). The specific shape of each target was held constant for each participant (e.g. the triangle was always the FT), but counterbalanced across participants. The participant was instructed to first foveate the FT, i.e. the triangular target in Fig 1. After the participant had kept fixation for a random duration of 200–500 ms (discouraging anticipatory saccades), an auditory signal (1kHz sine-wave beep, 60 ms) instructed the participant to saccade to the ST. The saccade was always in horizontal direction, either leftward or rightward in randomized order. The NT appeared midway between the ST and FT, above or below (randomized). The exact position of these targets relative to the screen’s center was varied (over a range of 27° horizontally and 20.6° vertically, flat distributions) in order to deter learning the exact location of the targets on the monitor. During the saccade, at on average 36 ± 8.3 ms (mean ± std) after saccade onset, one of the three targets was displaced, while the other two were removed from the display. The target displacement (-5, -3, -2, -1, -½, 0, ½, 1, 2, 3, or 5 degrees) was parallel or orthogonal to the saccade. The displaced target remained visible for 50 ms, 300 ms, or for about 1000 ms until a response was given, the ‘1000 ms’ condition. The time between saccade offset and the response was kept constant such that memory decay of the presaccadic scene was similar for the three viewing conditions. Together, this defined 792 trial types (i.e. 2 saccade directions, 3 targets, 2 NT locations, 11 displacement sizes, 2 displacement directions (parallel vs. orthogonal), and 3 viewing durations). For our first six participants, the 50 ms and 300 ms condition were randomly presented in the first three experimental sessions; the 1000 ms condition was tested in a separate session. For the other group of participants, the three viewing time conditions were fully mixed in all four sessions. No significant differences between both groups were found.

Participants gave their response using a mouse cursor (small crosshair) indicating the presaccadic position of the displaced target, which they confirmed by clicking the left mouse button. The cursor appeared always 300 ms after the displacement occurred. Participants performed each trial type 4 or 5 times. In case the saccade endpoint deviated more than 5° from the ST location, a red screen was shown for 1000 ms after a response was given. Eye blinks that triggered the target to jump were also followed with a red screen. If the participant did not know about which of the three targets to report, he or she had to shift the cursor to the left border of the display, before clicking the mouse button. Before the actual experiment started the participant completed a series of practice trials until s/he felt comfortable with the task.

### Data analysis

We performed offline data analyses in Matlab (The Mathworks, Nattick, MA). Trials in which the target displacement did not occur during the saccade (eye velocity < 50°/s for offline analysis) were discarded (14.6 ± 2.0%; mean ± SEM). Trials in which the postsaccadic target was not perceived (2.7 ± 0.7%) and trials with localization responses that were closest to a target other than the original position of the postsaccadic target were also discarded (3.7 ± 1.4%). We also discarded trials with a red screen (2.7± 0.6%). As a result, each participant completed on average 2427 ± 111 correct trials. Across participants, saccade duration was 50.7 ± 1.1 ms and saccade amplitude 14.0 ± 0.2°. There was no instruction on saccade reaction time. Average saccade latency, 273.7 ± 45.4 ms (mean ± SEM), was higher than usual, probably because of the memorization of the presaccadic positions (cf. [30]). The total duration that the targets were displayed before the saccade was on average 1200 ± 60 ms.

Data of four experimental configurations, that is a left/rightward saccade and NT above/below, were pooled by transforming them toward the single configuration shown Fig 1A, reducing the number of unique trial types to 198. Localization error was defined relative to the presaccadic target location, and was signed positive into the horizontal saccade direction and vertically upwards (see Fig 1A).

### Mixture model

We modeled the role of causal inference in the computations to obtain spatial constancy. The model has to explain the observed responses of each participant. Our principal model involves a statistically optimal mixture at the trial level of two possible causal structures on the signals available. This 2D model is developed here, formulated along the lines proposed in Körding et al. [11], to which we will frequently refer for further information. In the subsection ‘Alternative Models’ below we will introduce two variants of this model, also considered by Wozny et al.[10], involving at the trial level not a mixture of, but a choice between the two possible causal structures.

By estimating the causal relations between the various sources of information the brain attempts to determine whether two percepts belong together or need to be processed independently. More specifically, on each trial the task of the system is to estimate the presaccadic target position on the screen, denoted *s*, based on two sources of information, the memory-based remapped presaccadic position percept, denoted *m*, and the position percept of the postsaccadic visual stimulus, denoted *v*. Both entities are available with finite precision only (having some amount of noise) and are represented by probability distributions, which constitute the input to the causal inference model expounded below. First, we briefly describe how we modeled these probability distributions of the single source percepts *m* and *v*.

The distributions of both *m* and *v* are assumed to be independent 2D Gaussians. It can be expected that the variance of *m* has several sources, such as retinal noise during target encoding, remapping noise related to target updating, and noise due to memory decay. Some of the noise sources may be anisotropic (e.g. [9]). For simplicity, we do not model these sources but use a combined estimate $\sigma_{m}^{2}$ for each target position and allow anisotropy. Thus, $\sigma_{m}^{2}$ is estimated per target position, both for the parallel and orthogonal direction, resulting in 3x2 free parameters for *m*. For *v* we assume its variance to be isotropic, primarily determined by encoding noise. Intuitively, the shorter an object is viewed, the more noisy the position percept. Thus, $\sigma_{v}^{2}$ is estimated per viewing time condition (irrespective of target), resulting in 3 free parameters for *v*.

**Fig 6:**
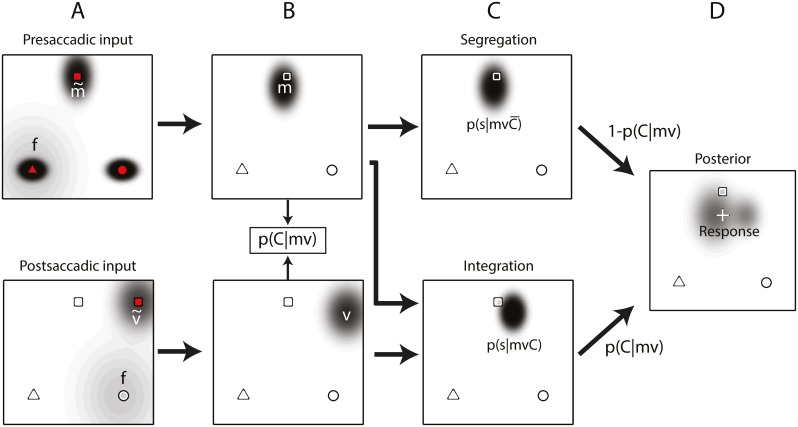
Mixture model. The presaccadic location of the NT (square) is reported after a transsaccadic displacement of 10° to the right. Objects in red represent visible targets; the white objects depict the veridical target locations. Representations of location estimates, modeled as 2D Gaussians, are shown as dark ellipses. (A) Before the saccade, all three objects are encoded with the foveal prior *f* (light grey blob) being centered at the triangle, the FT. After the saccade, the displaced target’s position and identity (NT here) are encoded with *f* now being centered at the saccade landing position. (B) Based on the NT’s presaccadic (*m*) and postsaccadic (*v*) representations, both biased by *f*, the probability of a single stable object, p(C|mv), is computed. In case *m* and *v* are unrelated the best solution is to segregate and ignore *v*. If *m* and *v* derive from the same object, the best solution is to integration all signals. (D) The two solutions in (C) are weighted according to the probability that *m* and v are related. The localization response follows from $p\left(s|mv\right)=p\left(s|mvC\right)\cdot p\left(C|mv\right)+p\left(s|mv\bar{C}\right)\cdot p\left(\bar{C}|mv\right)$.

It has further been suggested that participants localize visual targets towards the fovea (e.g. [31–33]). We modeled this foveal bias by including a prior, specified as an independent isotropic 2D Gaussian with variance $\sigma_{f}^{2}$, centered at FT for *m* and at the saccadic landing point for *v* (see Fig 6A), and by interpreting the percepts *m* and *v* as the results of an optimal Bayesian integration process of accurate sensory signals $\tilde{m}$ and $\tilde{v}$, respectively, with this prior. As a consequence, the center of *m* is not at the true target position, but shifted in the direction of FT by the fraction $\frac{\sigma_{m}^{2}}{\sigma_{f}^{2}}$ of the distance between these points (see Fig 6B). Similarly, the center of *v* shifts from the true target position in the direction of the saccade landing point by the fraction $\frac{\sigma_{v}^{2}}{\sigma_{f}^{2}}$ of the distance between these two points.

These single source distributions play an essential role in the mixture model, in which the evidence for target position *s* given memory information *m* and visual information *v* takes the form of a probability density function *p*(*s*|*mv*). Thus, *p*(*s*|*mv*) is the localization response, given estimates *m* and *v*. In order to determine this *p*(*s*|*mv*) in an optimal way, the system has to process correctly the probabilistic information available in *m* and *v*. That is, the system has to acknowledge that, while there is a direct relationship between *m* and *s* on each trial, this is not the case for *v* and *s*. Depending on the discrepancy between the two sources of information the system may either see no evidence for a displacement and consider the information *v* as relevant for the presaccadic position *s* to be reported (Fig 6C; integration), or it may take *v* to refer to a new visual object without a clear relationship with *s* (Fig 6C; segregation). In short, the system may distinguish two kinds of trials, requiring different forms of *p*(*s*|*mv*). In this probabilistic setting the optimal procedure for the system is not to choose per trial one of these forms, but to apply on any trial a mix of both, with the weight for each form equal to the estimated probability of it being the correct one given sources of information *m* and *v* (Fig 6D). Denoting the situation of a trial where both *m* and *v* derive directly from the presaccadic position *s* by *C* (common cause for *m* and *v*) and one where *v* derives from a different object (the displacement) by $\overset{-}{C}$(no common cause for *m* and *v*), this leads to a mixture model of the representation of *p*(*s*|*mv*)[11]:

$$p\left(s|mv\right)=p\left(s|mvC\right)\cdot p\left(C|mv\right)+\;p(s|mv\bar{C})\cdot p\left(\bar{C}|mv\right)$$ (1)

This model consists of three components: (i) *p*(*s*|*mvC*), the distribution of *s* given *m* and *v* when *v* is the sensory representation of the true position; (ii) $p(s|mv\overset{-}{C})$, the distribution of *s* given *m* and *v* when *v* does not represent the true position, but a displaced version of it; and (iii) *p*(*C*|*mv*), the probability that the current *m* and *v* are from a trial with common source, with $p\left(\bar{C}\text{|}mv\right)\;=\;1-p\left(C\text{|}mv\right)$ the complementary probability of a trial with *m* and *v* referring to different positions. We will now discuss the specification of these three components in turn.

#### (i) The distribution of s under the assumption of no displacement

In this situation both *m* and *v* are directly informative about the true position *s* and this is a case for the standard optimal integration model. By the laws of probability (“Bayes rule”) and assuming that *m* and *v* constitute two independent sources of information of a specific position *s* we obtain:

$$p\left(s|mvC\right)=\frac{p\left(mv|sC\right)\cdot p\left(s|C\right)}{p\left(mv|C\right)}=\;\frac{p\left(m|sC\right)\cdot p\left(v|sC\right)\cdot p\left(s|C\right)}{p\left(mv|C\right)}$$ (2)

Here, the denominator is a normalizing constant, independent of *s*, while the three factors of the numerator represent, respectively, the likelihoods of remapped position *m* and visual sensory information *v* given *s*, and the prior probability of *s*, the probability of *s* being at a certain spot of the screen independent of any sensory trial information, all of this in trials without a displacement. The first two are the independent 2D Gaussian *m* and *v* distributions described above, and the prior for *s* is taken to be of the same kind, centered at some point *π* of the screen and having anisotropic variance ($\sigma_{\pi x}^{2}$ and $\sigma_{\pi y}^{2}$)

#### (ii) The distribution of s under the assumption of a displacement

In this case, *m* still derives directly from the true position *s*, but *v* refers to a different position. Without any systematic relationship between this new position and *s* it is unclear how *v* can contribute to the estimation of *s*. The optimal procedure is then to not integrate and disregard *v*. In terms of probability distributions:

$$p\left(s|mv\bar{C}\right)=p(s|m\bar{C})=\;\frac{p\left(m|s\bar{C}\right)\cdot p\left(s|\bar{C}\right)}{p\left(m|\bar{C}\right)}$$ (3)

Actually, the distinction between *C* and $\overset{-}{C}$ trials has only to do with the role of the information *v* and there is no reason why the likelihood of *m* or the prior for *s* would be different for the two kinds of trials. That is, these distributions can be taken identical to their counterparts in the *C* trials described in (i) above and the consequence is that the specification of $p(s|mv\overset{-}{C})$ coincides with that of *p*(*s*|*mvC*) apart from deleting the contribution made by *v*.

#### (iii) The probability of the trial having vs. not having a displacement

The data *m* and *v* on a specific trial are also informative for assigning optimal relative weights to the estimate for *s* obtained under the assumption of no displacement (case (i) above) and the estimate under the assumption of a displacement (case (ii) above). Intuitively, the larger the discrepancy between *m* and *v* of a given trial, the more evidence that they are not emanating from the same source, i.e., the more evidence for a displacement trial. This can again be made precise by the laws of probability, including Bayes rule:

$$p\left(C|mv\right)=\frac{p(mv|C)\cdot p\left(C\right)}{p\left(mv\right)}=\;\frac{p(mv|C)\cdot p\left(C\right)}{p(mv\left|C)\cdot p\left(C\right)\;+\;p(mv\right|\bar{C})\cdot p\left(\bar{C}\right)}$$ (4)

The latter equation expresses how the probability of the trial outcomes *m* and *v* having a common source (no displacement) depends on a prior probability, independent of trial information, for common source trials, *p*(*C*), and on the likelihoods of the obtained *m*-*v* combination for no-displacement (common source) and displacement trials, *p*(*mv*|*C*) and $p(mv|\overset{-}{C})$, respectively. The first of these, the prior common source probability *p*(*C*) is simply taken as a free parameter *p*_*c*_ in the model, with $p(\overset{-}{C})\;=\;1-p_{C}$.

As for the *m*-*v* likelihood in no-displacement trials, this can be mathematically obtained as the weighted average across all possible *s* positions [11]. Assuming independence, this can be done for two orthogonal directions separately. For the parallel (i.e. horizontal) direction, indexed by *x*, it follows:

$$p\left(m_{x}v_{x}|C\right)=\int p\left(m_{x}v_{x}|s_{x}\right)p\left(s_{x}\right)ds_{x}=\int p\left(m_{x}|s_{x}\right)p\left(v_{x}|s_{x}\right)p\left(s_{x}\right)ds_{x}$$ (5)

Given the Gaussian assumptions for *p*(*m*_*x*_|*s*_*x*_), *p*(*v*_*x*_|*s*_*x*_), and *p*(*s*_*x*_), this integral has an analytic solution (see [11]):

$$p\left(m_{x}v_{x}|C\right)=\frac{1}{2\pi \sqrt{\sigma_{mx}^{2}\sigma_{v}^{2}+\sigma_{mx}^{2}\sigma_{\pi x}^{2}+\sigma_{v}^{2}\sigma_{\pi x}^{2}}}\times exp\left[-\frac{1}{2}\left(\frac{{\left(m_{x}-v_{x}\right)}^{2}\sigma_{\pi x}^{2}\;+\;{\left(m_{x}-\pi_{x}\right)}^{2}\sigma_{v}^{2}\;+\;{\left(v_{x}-\pi_{x}\right)}^{2}\sigma_{mx}^{2}}{\sigma_{mx}^{2}\sigma_{v}^{2}+\sigma_{mx}^{2}\sigma_{\pi x}^{2}+\sigma_{v}^{2}\sigma_{\pi x}^{2}}\right)\right]$$ (6)

An analogous equation can be derived for the *m-v* likelihood in the orthogonal (i.e.vertical) direction (*y*), yielding *p*(*m*_*y*_*v*_*y*_|*C*). The 2-D likelihood *p*(*mv*|*C*) is then obtained as the product of these horizontal and vertical likelihoods.

As to the *m*-*v* likelihood in displacement trials, we note that *m* and *v* are regarded independent, not connected by a common *s*, and thus their weighted averages across *s* positions have to be computed independently [11]. This amounts to

$$p\left(m_{x}v_{x}|\bar{C}\right)=p\left(m_{x}|\bar{C}\right)\cdot p\left(v_{x}|\bar{C}\right)=\int p\left(m_{x}|s_{x}\right)p\left(s_{x}\right)ds_{x}\cdot \int p\left(v_{x}|s_{x}\right)p\left(s_{x}\right)ds_{x}$$ (7)

which given our Gaussian assumptions has again an analytical solution, now as a product of two Gaussians [11]:

$$p\left(m_{x}v_{x}|\bar{C}\right)=\frac{1}{2\pi \sqrt{\left(\sigma_{mx}^{2}+\sigma_{\pi x}^{2}\right)\left(\sigma_{v}^{2}+\sigma_{\pi x}^{2}\right)}}exp\left[-\frac{1}{2}\left(\frac{{\left(m_{x}-\pi_{x}\right)}^{2}}{\sigma_{mx}^{2}+\sigma_{\pi x}^{2}}+\frac{{\left(v_{x}-\pi_{x}\right)}^{2}}{\sigma_{v}^{2}+\sigma_{\pi x}^{2}}\right)\right]$$ (8)

In combination with the analogous expression for the vertical direction, we achieve $p\left(mv\text{|}\bar{C}\right)\;=\;p\left(m_{x}v_{x}\text{|}\bar{C}\right)\cdot p\left(m_{y}v_{y}\text{|}\bar{C}\right)$.

### Model fitting and evaluation

The model contains 15 free parameters to fit 2D localization data from 198 different conditions: 3 target positions (FT, ST, NT) x 11 displacement sizes (-5° to 5°) x 2 displacement directions (parallel, orthogonal) x 3 viewing times (50, 300, 1000 ms). Six parameters are used to estimate *m*; three parameters are used for *v* (see Mixure Model). The remaining six parameters describe the priors: one for the foveal bias ($\sigma_{f}^{2}$), four for the *x*,*y* position (allocentric) and anisotropic variance of *π*, and finally one for the general expectation of perceiving a common source (*p*_*c*_). These parameters were fit to all localization responses simultaneously for each participant (mean: 2589 data points) using Matlab’s *fminsearch* with 1000 searches (random initial parameter values) per participant. In every iteration of the search process, each condition was simulated 10000 times. These distributions were then compared (using 0.1° bins) to the actual localization data in order to estimate the likelihood of the data given the model. Across iterations, the parameters were adjusted until an optimal fit was reached, i.e., the loglikelihood was maximized.

### Alternative models

The above mixture model assumes a causal inference process that is fully statistically optimal. Of course, it is questionable whether the brain can attain such absolute optimality. To test for this, we additionally fitted two variants of the mixture model, suboptimal in the statistical sense, following proposals by Wozny et al. [10]. These two alternative models use the same ingredients as the mixture model, but differ by the response rule applied. On each trial, given an estimate of *p*(*C*|*mv*), the common-cause probability of the trial, this probability is not used for weighting the common-cause, *p*(*s*|*mvC*), and no-common-cause, $p\left(s|mv\bar{C}\right),\;$ distributions of the target as in Eq (1), but for choosing one of these. While making such a forced choice is not optimal, the choice itself can be made in an optimal way and this constitutes the first alternative model (referred to as model selection): per trial just choose the more likely causal structure, i.e., if *p*(*C*|*mv*)>0.5, choose *p*(*s*|*mvC*), otherwise choose $p\left(s|mv\bar{C}\right)$. The second alternative model (referred to as probability matching) amounts to one more step away from optimality: here the choice between the two causal structures is again guided by the common-cause probability of the trial, but now according to the principle of probability matching: with probability equal to *p*(*C*|*mv*) choose *p*(*s*|*mvC*) and with complementary probability $p\left(\bar{C}|mv\right)$ choose $p\left(s|mv\bar{C}\right)$.

The model fitting procedure for the two alternative models is identical to the one for the mixture model described above (e.g. same number of free parameters) and log-likelihoods are compared to determine which model describes the data best for each individual participant.
